# Supplementary material for: Content-rich biological network constructed by mining PubMed abstracts
Source: BMC Bioinformatics. 2004 Oct 8;5:147. doi: 10.1186/1471-2105-5-147 (PMC528731; doi:10.1186/1471-2105-5-147)
Supplement: Additional File 2 — The original results of the above study (non-essential files are deleted to keep the file size under the limit set by BMC bioinformatics). [file 1471-2105-5-147-S2.bz2 › chilibotAdditionalFile2/dip50/582ID9571026E10950/html/GHR_GH1.html]

 


 **GHR** and **GH1** 
  
Found 60 abstracts in PubMed, retrieved 50.  
 

 What does Google say? 
 PDF only 
| .edu only 

---

**Interactive relationship** (e.g. stimulation, inhibition, etc)

**Neutral relationship**- Northern blotting of mRNA for somatotropin  [ **GH1** ]  receptor  [ **GHR** ]  showed expression of somatotropin  [ **GH1** ]  receptor  [ **GHR** ]  mRNA transcripts in whole ovary 4.7 and 4.4 kb, CL 4.7 and 4.4 kb, and liver 4.4 kb.  Ref: 8318577 Biol Reprod, 1993
- Partial purification of somatotropin  [ **GH1** ]  receptors from pig liver they arise from a single somatotropin  [ **GH1** ]  receptor  [ **GHR** ]  messenger RNA transcript.  Ref: 1459902 J Anim Sci, 1992
- Other adult and fetal bovine tissues expressed 1B somatotropin  [ **GH1** ]  receptor  [ **GHR** ]  mRNA with a predicted protein sequence that was similar to the 1A somatotropin  [ **GH1** ]  receptor  [ **GHR** ] .  Ref: 9710756 J Dairy Sci, 1998
- Pituitary growth hormone  [ **GH1** ]  GH acts as a growth promoter in a wide range of tissues after binding to its specific GH receptor  [ **GHR** ]   **GHR**  or to a cytosolic circulating GH binding protein  [ **GHR** ]  GHBP  [ **GHR** ] .  Ref: 7789615 Mol Cell Endocrinol, 1995
- In summary, ovarian derived GH N  [ **GH1** ]  and PL A B synthesis correlates well with the established local cascade of GHRH, GHRH receptor, GH receptor  [ **GHR** ] , IGF I, and IGF I receptor as a putative para autocrine regulator of ovarian reproductive function.  Ref: 9329365 J Clin Endocrinol Metab, 1997
- We have very recently reported a child with short stature and a mutant GH caused by a single missense mutation in the GH 1  [ **GH1** ]  gene, which itself cannot transduce the GH signal to the cells but can blunt the action of wild type GH by virtue of its greater affinity for the GH binding protein  [ **GHR** ]  GHBP  [ **GHR** ]  GH receptor  [ **GHR** ] .  Ref: 9076339 Endocr J, 1996
- These results indicate that the D112G mutation in the GH 1  [ **GH1** ]  gene causes production of bioinactive GH, which prevents dimerization of  **GHR**  and is therefore responsible for the patient s short stature.  Ref: 9276733 J Clin Invest, 1997

**Non-interactive relationship** (e.g. studied together, co-existance, homology, etc.)

- As indicated by immunoperoxidase staining, CL expressed immunoreactive somatotropin  [ **GH1** ]  receptor  [ **GHR** ]  positive stain.  Ref: 8318577 Biol Reprod, 1993
- In liver, treatment did not alter the abundance of mRNA for the somatotropin  [ **GH1** ]  receptor  [ **GHR** ]  or the number of free binding sites for somatotropin  [ **GH1** ] .  Ref: 8598398 J Dairy Sci, 1995
- Pituitary growth hormone  [ **GH1** ]  GH, prolactin PRL, placental lactogen PL, insulin like growth factor 1 IGF 1 and GH binding protein  [ **GHR** ]  GHBP  [ **GHR** ]  in plasma were determined in 12 women with gestational diabetes mellitus GDM and in 12 healthy pregnant women during a breakfast meal tolerance test.  Ref: 8001865 Gynecol Obstet Invest, 1994
- The ability of human tonsillar lymphoid cells to express growth hormone receptor  [ **GHR** ]  hGH N  [ **GH1** ]  R was analyzed by flow cytometry.  Ref: 9814603 Dev Immunol, 1998
- The objective was to characterize P1 activity by measuring the amount of growth hormone receptor  [ **GHR** ]  1A mRNA in liver of dairy cows at different stages of lactation as well as after administration of recombinant bovine somatotropin  [ **GH1** ]  rbST.  Ref: 10509249 J Dairy Sci, 1999
- On the basis of solution hybridization nuclease protection, the mRNA for somatotropin  [ **GH1** ]  receptor  [ **GHR** ]  was found in greatest abundance in CL and large luteal cells and was nearly undetectable in small luteal cells or follicles class 5 mm.  Ref: 8318577 Biol Reprod, 1993
- Studies have shown that bovine placental lactogen bPL has partial somatogenic activity in vivo even though binding results clearly indicate bPL does not cause homodimerization of the bovine somatotropin  [ **GH1** ]  receptor  [ **GHR** ]  bST R.  Ref: 10622285 Life Sci, 1999
- Ovarian stroma, connective tissue, endothelium, and erythrocytes did not express somatotropin  [ **GH1** ]  receptor  [ **GHR** ]  negative stain.  Ref: 8318577 Biol Reprod, 1993
- We present a computer derived three dimensional model of the IL 6 IL 6 receptor complex based on the structure of the human somatotropin  [ **GH1** ]  human somatotropin  [ **GH1** ]  receptor  [ **GHR** ]  complex.  Ref: 8706672 Eur J Biochem, 1996
- Alternative sizes of mRNA for somatotropin  [ **GH1** ]  receptor  [ **GHR** ]  suggest novel mRNA processing in the bovine ovary.  Ref: 8318577 Biol Reprod, 1993
- Our objective, therefore, was to sequence a cDNA for the 1B somatotropin  [ **GH1** ]  receptor  [ **GHR** ]  and to analyze bovine tissues for expression of 1A and 1B somatotropin  [ **GH1** ]  receptor  [ **GHR** ]  mRNA.  Ref: 9710756 J Dairy Sci, 1998
- We therefore determined the expression of growth hormone GH receptor  [ **GHR** ]   **GHR** , and insulin like growth factors I and II IGF I and IGF II mRNA in liver and skeletal muscle longissimus of neonatal pigs given daily intramuscular injections of either recombinant porcine GH 1  [ **GH1** ]  mg kg body wt.  Ref: 10749770 Am J Physiol Regul Integr Comp Physiol, 2000
- Immunohistochemical and nucleic acid analysis of somatotropin  [ **GH1** ]  receptor  [ **GHR** ]  populations in the bovine ovary.  Ref: 8318577 Biol Reprod, 1993
- Pituitary growth hormone  [ **GH1** ]  GH is essential for postnatal growth in animal, it regulates numerous cellular functions by direct effect on it s receptor  **GHr**  in many different tissues.  Ref: 12096625 Yi Chuan Xue Bao, 2002
- Expression of growth hormone receptor  [ **GHR** ]  1A messenger ribonucleic acid in liver of dairy cows during lactation and after administration of recombinant bovine somatotropin  [ **GH1** ] .  Ref: 10509249 J Dairy Sci, 1999
- The alignment of its amino acid sequence with those of other members of this family human somatotropin  [ **GH1** ]  receptor  [ **GHR** ]  murine IL 3 receptor beta and human IL 2 receptor beta has suggested that amino acids included in two SSFY repeats found in each of its hematopoietin receptor domains, contribute to the binding of the ligand.  Ref: 8706672 Eur J Biochem, 1996
- Although fetal growth is generally considered to be independent of pituitary growth hormone  [ **GH1** ]  GH, it is possible that pituitary GH plays a modulatory role in organ development or that a GH like substance of non pituitary origin may influence fetal growth through the GH receptor  [ **GHR** ] .  Ref: 1618149 Development, 1992
- Ovaries were analyzed for somatotropin  [ **GH1** ]  receptor  [ **GHR** ]  protein and mRNA through use of immunohistochemistry, solution hybridization nuclease protection, Northern blotting, and reverse transcriptase polymerase chain reaction RT PCR.  Ref: 8318577 Biol Reprod, 1993
- In summary, somatotropin  [ **GH1** ]  receptor  [ **GHR** ]  both immunoreactive protein and mRNA is found primarily in the large luteal cell, and lesser amounts of the expressed receptor or its message are found in the follicle.  Ref: 8318577 Biol Reprod, 1993
- Continuous long term porcine somatotropin  [ **GH1** ]  pST injections daily for 6 to 7 wk increased GHBP  [ **GHR** ]  activity P less than.  Ref: 1399889 J Anim Sci, 1992
- Evaluation of the somatogenic activity of bovine placental lactogen with cell lines transfected with the bovine somatotropin  [ **GH1** ]  receptor  [ **GHR** ] .  Ref: 10622285 Life Sci, 1999
- and RT PCR amplified a single amino acid coding region for somatotropin  [ **GH1** ]  receptor  [ **GHR** ]  in CL and liver.  Ref: 8318577 Biol Reprod, 1993
- Northern blot analysis revealed that these proteins arise by posttranslational modification of a single 4.2 kilobase somatotropin  [ **GH1** ]  receptor  [ **GHR** ]  messenger RNA transcript.  Ref: 1459902 J Anim Sci, 1992
- Somatotropin  [ **GH1** ]  dependent regulation of IGF I was only observed in liver, where the greatest somatotropin  [ **GH1** ]  receptor  [ **GHR** ]  mRNA concentration was found.  Ref: 9834461 J Endocrinol, 1998
- The somatotropin  [ **GH1** ]  receptor  [ **GHR** ]  mRNA is controlled by at least two different gene promoters that generate two variants with different exon 1 sequences 1A and 1B.  Ref: 9710756 J Dairy Sci, 1998
- The location of 1A and 1B somatotropin  [ **GH1** ]  receptor  [ **GHR** ]  mRNA within cattle tissues and, hence, the tissue specificity of the 1A and 1B promoters are unknown.  Ref: 9710756 J Dairy Sci, 1998
- Expression of somatotropin  [ **GH1** ]  receptor  [ **GHR** ]  messenger ribonucleic acid in bovine tissues.  Ref: 9710756 J Dairy Sci, 1998
- Most follicles 1 5 mm, after fixation were negative for somatotropin  [ **GH1** ]  receptor  [ **GHR** ] .  Ref: 8318577 Biol Reprod, 1993
- Within the CL, somatotropin  [ **GH1** ]  receptor  [ **GHR** ]  protein was expressed primarily in large luteal cells whereas small luteal cells were negative.  Ref: 8318577 Biol Reprod, 1993
- We concluded that 1A somatotropin  [ **GH1** ]  receptor  [ **GHR** ]  mRNA is specific to adult bovine liver.  Ref: 9710756 J Dairy Sci, 1998
- In addition, the cDNA sequence of the 1B somatotropin  [ **GH1** ]  receptor  [ **GHR** ]  has not been determined.  Ref: 9710756 J Dairy Sci, 1998
- The mRNAs for somatotropin  [ **GH1** ]  receptor  [ **GHR** ] , IGFs I and II, IGFBP 2 and pregnancy associated glycoprotein a marker of trophoblast tissue were analyzed by Northern blotting or ribonuclease protection assay.  Ref: 9834461 J Endocrinol, 1998
